# Amplification of *KRAS* and its heterogeneity in non-Asian gastric adenocarcinomas

**DOI:** 10.1186/s12885-020-06996-x

**Published:** 2020-06-22

**Authors:** Jan Rehkaemper, Michael Korenkov, Alexander Quaas, Josef Rueschoff, Aylin Pamuk, Thomas Zander, Axel M. Hillmer, Reinhard Buettner, Arnulf Heinrich Hoelscher, Christiane Josephine Bruns, Heike Loeser, Hakan Alakus, Birgid Schoemig-Markiefka

**Affiliations:** 1 Institute of Pathology, University Hospital Cologne, Cologne, Germany; 2 Department of General, Visceral and Cancer Surgery, University Hospital Cologne, Cologne, Germany; 3 Institute of Pathology, Nordhessen and Targos Molecular Pathology GmbH, Kassel, Germany; 4 Department of Internal Medicine I, University Hospital Cologne, Cologne, Germany; 5 Center for Esophageal and Gastric Surgery, AGAPLESION Markus Krankenhaus, Frankfurt, Germany

**Keywords:** *KRAS* amplification, Gastric adenocarcinoma, Prognosis, Fluorescence-in-situ-hybridization (FISH), Heterogeneity

## Abstract

**Background:**

Gastric cancer is one of the deadliest cancer entities worldwide. While surgery is the only curative treatment option in early tumors, for locally advanced and metastatic patients further therapeutic targets are needed. Several studies not only reported mutations but also amplifications of the *KRAS* locus in different cancer entities. More recently, *KRAS* amplification was discussed as a new therapeutic target. Little is known about the (prognostic) relevance and (heterogenic) distribution of *KRAS* amplification in gastric adenocarcinomas, especially in Non-Asian patients.

**Methods:**

Amplification of the *KRAS* locus and corresponding protein expression was analyzed in 582 gastric adenocarcinomas employing fluorescence in-situ hybridization (FISH) and immunohistochemistry. Amplification status was correlated with clinico-pathological features, clinical outcome and molecular tumor data including a correlation to the TCGA subtypes of gastric carcinoma.

**Results:**

*KRAS* amplification was detected in 27 out of 470 analysable tumors (5.7%) and correlated with protein expression of KRAS in all amplified tumors. Within the *KRAS* amplified gastric tumors 14/27 (51.9%) showed a heterogeneous distribution with also *KRAS* non-amplified tumor parts. According to TCGA 24 tumors (88.8%) were related to chromosomal instable tumors (CIN). The survival analysis of the entire patient cohort did not show any difference in overall survival in dependence on the *KRAS* status. However, a significant survival difference with a worse outcome for patients with *KRAS* amplified tumors was identified when analysing patients without neoadjuvant pre-treatment.

**Conclusions:**

We confirm the unfavorable prognosis of *KRAS* amplified tumors reported by other studies in (Asian) patient groups, at least in patients without neoadjuvant pre-treatment. Within *KRAS* amplified tumors we revealed intratumoral heterogeneity that may define a (more aggressive) tumor cell population which is more frequently observed in patients with lymph node metastases. Despite the heterogeneous distribution of *KRAS* amplified tumor clones, *KRAS* amplified locally advanced or metastasized gastric adenocarcinomas represent a therapeutically highly relevant tumor subgroup.

## Background

Gastric cancer is one of the most commonly diagnosed cancers, and in both sexes combined the third leading cause of cancer-related deaths worldwide [1, 2]. Prominent differences in age-standardized incidence are observed in different parts of the world ranging from 32.1% in eastern Asia to 8,2% in Western Europe [2]. Among sporadic (distal) gastric adenocarcinomas *Helicobacter pylori* infection remains the most important cause which can partly reflect the different incidence rates worldwide [3, 4].

In advanced tumor stages and especially in metastatic patients the overall survival is still poor with a median overall survival of less than 1 year in the latter [5]. Therefore, molecular targets need to be identified providing further therapeutic options.

The only molecular alteration that is currently used therapeutically in gastric cancer is Her2/neu. The ToGa (Trastuzumab for Gastric Cancer) trial demonstrated improved overall survival in patients with Her2/neu amplified tumors who received trastuzumab in addition to standard chemotherapy in comparison to chemotherapy alone [6]. Within primary tumors intratumoral heterogeneity has been demonstrated in up to 33% and between those and distant metastasis in 11% [7]. Therefore, heterogeneity of gene amplifications within primary gastric adenocarcinomas is not uncommon.

The Cancer Genome Atlas research network (TCGA) has performed full genomic profiling of 259 primary gastric adenocarcinomas and reported four main molecular tumor subtypes: tumors positive for Epstein-Barr virus (EBV), microsatellite unstable tumors (MSI), genomically stable tumors (GS) and tumors with chromosomal instability (CIN) [8].

While in the past the mutational status of *KRAS* was of great interest, recent studies focused on high-level amplification of *KRAS*. In several cancer entities amplification of *KRAS* has been reported and was associated with poor clinical outcome in most cases [9, 10]. Amplifications occurred mutually exclusive with mutations in *KRAS* and were also demonstrated to be activating with marked overexpression of KRAS protein [8, 11]. The extent of KRAS amplification is only known from large Asian patient populations. To what extent these results can be transferred to a Caucasian patient collective is still unclear. Recently, a study could show a possible individualized therapy option for *KRAS* amplified gastric cancer [11].

In the present study we analyzed the tumor tissue of a large cohort of 582 Caucasian patients with gastric adenocarcinomas. We employed immunohistochemistry (IHC) and fluorescence in-situ hybridization (FISH) as sensitive and well established diagnostic tools to detect amplification and protein expression of *KRAS* and focused on its intratumoral heterogeneity. Amplification status was then correlated with clinico-pathological features, clinical outcome and molecular tumor data including a correlation to the TCGA subtypes of gastric carcinoma.

## Methods

### Statistical analysis

Patient data was prospectively abstracted into a database. Interdependences between staining, tumor characteristics and clinical data were compared with the use of Pearson’s chi-squared test and Fisher’s exact test and illustrated by cross-tables. Overall survival was evaluated from the date of surgery to death. Kaplan-Meier curves were generated and compared using the log-rank test. Data on patients with no event or lost follow up were censored at the last seen date. Multivariate analysis for prognostic factors was performed using the Cox regression model. A two-sided *p*-value < 0.05 was considered statistically significant. SPSS package version 25 (IBM, Armonk, New York) was used for all statistical analyses.

### Surgery

Standardized surgical treatment included subtotal distal or total gastrectomy extended with transhiatal resection of the oesophagus in case of an adenocarcinoma of the esophagogastric junction (Siewert 2 or 3), and a systematic D2 lymphadenectomy with the goal of complete resection (R0). Roux-en-Y jejunal loop obstruction with gastrojejunostomy was considered the method of choice in the reconstruction procedures. Patients with advanced gastric adenocarcinoma (cT3, cNx, M0) received either chemoradiation or chemotherapy.

### Cohort

Tumor samples of 582 patients with gastric adenocarcinoma were considered for analysis. According to the TCGA [8] and the WHO classification of tumors (Digestive System Tumours, 5th edition, 2019) tumors were classified into four subgroups: (1) Those tumors positive for EBV (using EBER-ISH), (2) Tumors with MSI (using immunohistochemistry for MLH1, PMS2, MSH2, MSH6 and/or MSI testing), (3) Genomically stable tumors (GS) with poorly cohesive type morphology and/or *RHOA* mutations and/or E-cadherin loss, (4) Tumors with chromosomal instability (CIN) with intestinal type morphology and *HER2* amplification/overexpression and/or *TP53* mutations.

### Tissue microarray (TMA)

For TMA one tissue core from each tumor sample was punched out and transferred into a TMA recipient block. TMA construction was performed as previously described [12, 13]. In brief, tissue cylinders with a diameter of 1.2 mm each were punched from selected tumor tissue blocks using a self-constructed semi-automated precision instrument and embedded in empty recipient paraffin blocks.

### Immunohistochemistry

Immunohistochemistry (IHC) was performed on TMA slides. The KRAS antibody (mouse monoclonal primary antibody, clone 9.13, Thermo Fisher) was stained on the automated Ventana/Benchmark slide stainer. Expression of KRAS in the cytoplasm of carcinoma cells was assessed according to the following criteria: negative or weak staining in < 5% of tumor cells (score 0); weak staining ≥5–20% of tumor cells (score 1); moderate to strong staining in ≥20% (score 2). The evaluation of immunohistochemical expression was assessed manually by two pathologists (A.Q. and J.R.). Discrepant results, which occurred only in a small number of samples, were resolved by consensus review.

### Fluorescence in-situ hybridization (FISH)

Fluorescence in-situ hybridization (FISH) for the evaluation of the *KRAS* gene amplification status was performed on TMA and large scale slides with the Zytolight SPEC KRAS/CEN 12 Dual Color Probe (Zytomed, Systems GmbH, Germany) according to the manufacturers’ protocol. Sample processing was performed as previously described [14]. Tumor tissue was scanned for amplification spots and heterogeneity using a 63x objective (DM5500 fluorescent microscope; Leica). Samples were evaluated as “*KRAS* amplified” with a ratio greater than 3 between *KRAS* gene and chromosome 12 centromere signals following previous studies [15].

## Results

### Clinico-pathological and patient characteristics

The entire patient cohort consisting of 582 patients with gastric adenocarcinoma were considered for analysis. In total there were 470 analysable cases for *KRAS* (80.8%) on the TMA (Table 1). Reasons for missing data included the lack of tissue samples or the absence of unequivocal cancer tissue on the TMA spot. Neoadjuvant treatment consisting of either chemo-radiation or chemotherapy was administered in 198 patients (35.4%). The median follow-up for patients still alive was 26 months with a calculated 5-year survival rate of 38.4%.

**Table 1:** *KRAS* amplification and clinico-pathological characteristics

		*KRAS* amplification						
		total (*n* = 470)		negative (*n* = 443)		positive (*n* = 27)		
		n	%	n	%	n	%	*p*-value
sex	male	295	62.8%	275	58.5%	20	4.3%	0.528
	female	145	30.9%	138	29.4%	7	1.5%	
	n.a.	30	6.4%	30	6.4%	0	0.0%	
agegroup	< 50	42	8.9%	41	8.7%	1	0.2%	0.339
	> 50	331	70.4%	305	64.9%	26	5.5%	
	not available	97	20.6%	97	20.6%	0	0.0%	
tumor stage	T1	65	13.8%	62	13.2%	3	0.6%	0.830
	T2	147	31.3%	136	28.9%	11	2.3%	
	T3	166	35.3%	157	33.4%	9	1.9%	
	T4	67	14.3%	63	13.4%	4	0.9%	
	not available	25	5.3%	25	5.3%	0	0.0%	
nodal stage	N0	165	35.1%	160	34.0%	5	1.1%	0.024
	N1	105	22.3%	100	21.3%	5	1.1%	
	N2	78	16.6%	71	15.1%	7	1.5%	
	N3	76	16.2%	66	14.0%	10	2.1%	
	not available	46	9.8%	46	9.8%	0	0.0%	
localisation	proximal	205	43.6%	186	39.6%	19	4.0%	0.092
	body	70	14.9%	69	14.7%	1	0.2%	
	distal	98	20.9%	91	19.4%	7	1.5%	
	not available	97	20.6%	97	20.6%	0	0.0%	

### *KRAS* amplification

**Fig. 1:**
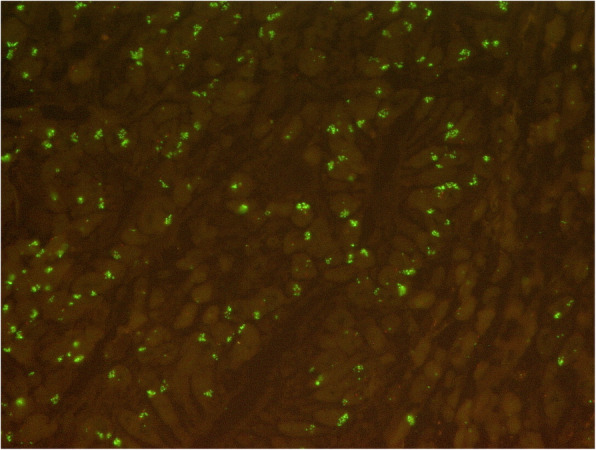
Primary gastric adenocarcinoma with *KRAS* amplification (*KRAS* FISH, 63x). Green cluster signals: *KRAS* locus (=amplification). Red signals: Centromer region of chromosome 12

*KRAS* amplifications were observed in 27 patients (5.7%) (for example see Fig. 1). The amplification profile for *KRAS* was not affected by the administration of neoadjuvant treatment. Within the group of patients undergoing primary surgery and the group of patients receiving neoadjuvant treatment first, 18 (6.0%) and 9 (6.3%) patients showed *KRAS* amplification respectively (*p* = 0.826). *KRAS* amplifications were not associated with sex (*p* = 0.528), age group (*p* = 0.339), tumor stage (*p* = 0.830) and localisation (a tendency toward proximal localization can be found) (*p* = 0.092). Nodal positive patients (pN+) were significantly correlated with a higher frequency of *KRAS* amplification (*p* = 0.024) (Table 1).

The analysis of *KRAS* amplification in relation to the molecular classification of gastric adenocarcinoma (according to TCGA) revealed that 24 (88.8%) were related to tumors with chromosomal instability (CIN) and 3 (11.1%) were related to genomically stable (GS) tumors (*p* = 0.282) (Table 2). No EBV or MSI-related gastric tumors harboured *KRAS* amplification.

**Table 2:** *KRAS* amplification and molecular subtype according to TCGA. CIN (chromsomal instable tumors); GN (genomically stable tumors); MSI (microsatellite instable tumors); EBV (Epstein-Barr virus-related tumors)

		*KRAS* amplification						
		total (*n* = 470)		negative (*n* = 443)		positive (*n* = 27)		*p*-value
TCGA	CIN	350	74.5%	326	73.6%	24	88.9%	0.282
	GN	58	12.3%	55	12.4%	3	11.1%	
	MSI	36	7.7%	36	8.1%	0	0.0%	
	EBV	24	5.1%	24	5.4%	0	0.0%	
	not available	2	0.4%	2	0.5%	0	0.0%	

### *KRAS* and prognosis

**Fig. 2:**
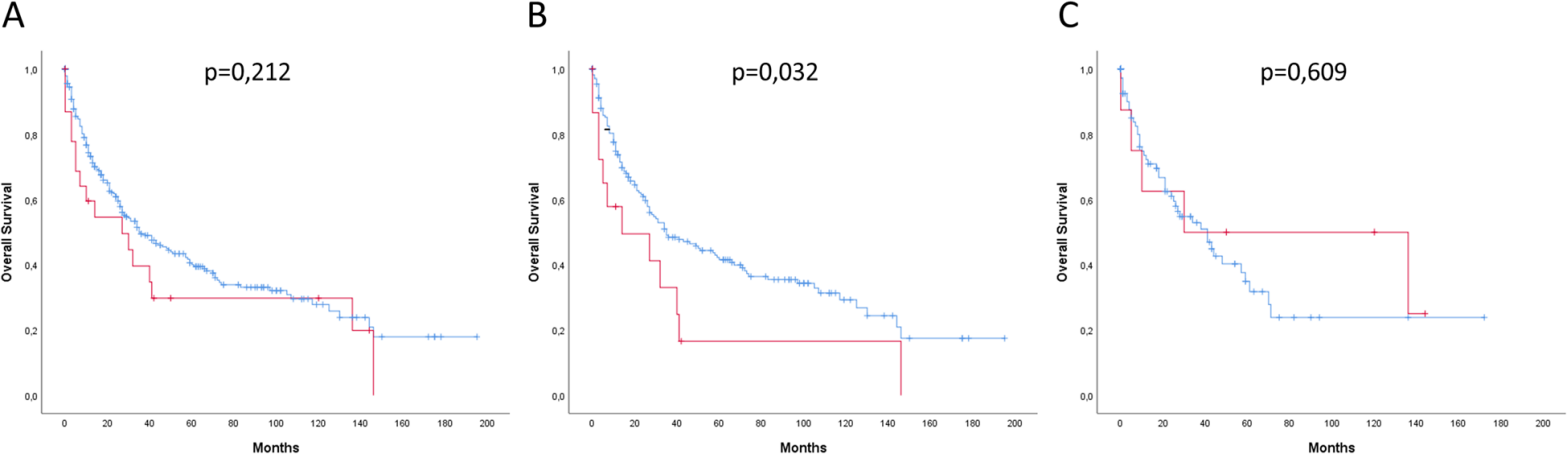
Kaplan-Meier survival analysis comparing patients with *KRAS* amplified gastric adenocarcinomas (red lines) and patients without *KRAS* amplified gastric adenocarcinomas (blue lines). **a** Whole patient cohort including neoadjuvant and primary-resected tumors (*n* = 347). *KRAS* amplified gastric adenocarcinomas (red line; *n* = 23) and adenocarcinomas without *KRAS* amplification (blue line; *n* = 324). **b** Patients without neoadjuvant pretreatment (primary resected tumors) with *KRAS* amplified tumors (red line; *n* = 16) in comparison to patients without *KRAS* amplified tumors (blue line; *n* = 229). *KRAS* amplified tumors in the primary resected cohort show a statistically significant worse prognosis in comparison to non-*KRAS* amplified tumors (*p* = 0.032). **c** Patients with preoperative neoadjuvant treatment with *KRAS* amplified tumos (red line; *n* = 7) in comparison to patients without *KRAS* amplified tumors (*n* = 94; blue line)

The survival analysis of the entire patient cohort did not show any difference in overall survival in dependence on the *KRAS* status (*p* = 0.212). However, a significant survival difference with a worse outcome for patients with *KRAS* amplification was identified when analysing patients without a neoadjuvant pre-treatment (primary resected). The median overall survival (OS) for *KRAS* wild-type patients was 35 months (95% CI: 19.2–50.8 months) compared to 16 months (95% CI: 0–46.36 months) for *KRAS* amplified patients (*p* = 0.032) (Fig. 2).

A multivariate cox proportional hazard model revealed that *KRAS* amplifications cannot be considered as an independent prognostic marker. The poor prognosis is driven by an accumulation of lympho-nodal metastatic patients.

### *KRAS* and heterogeneity

**Fig. 3:**
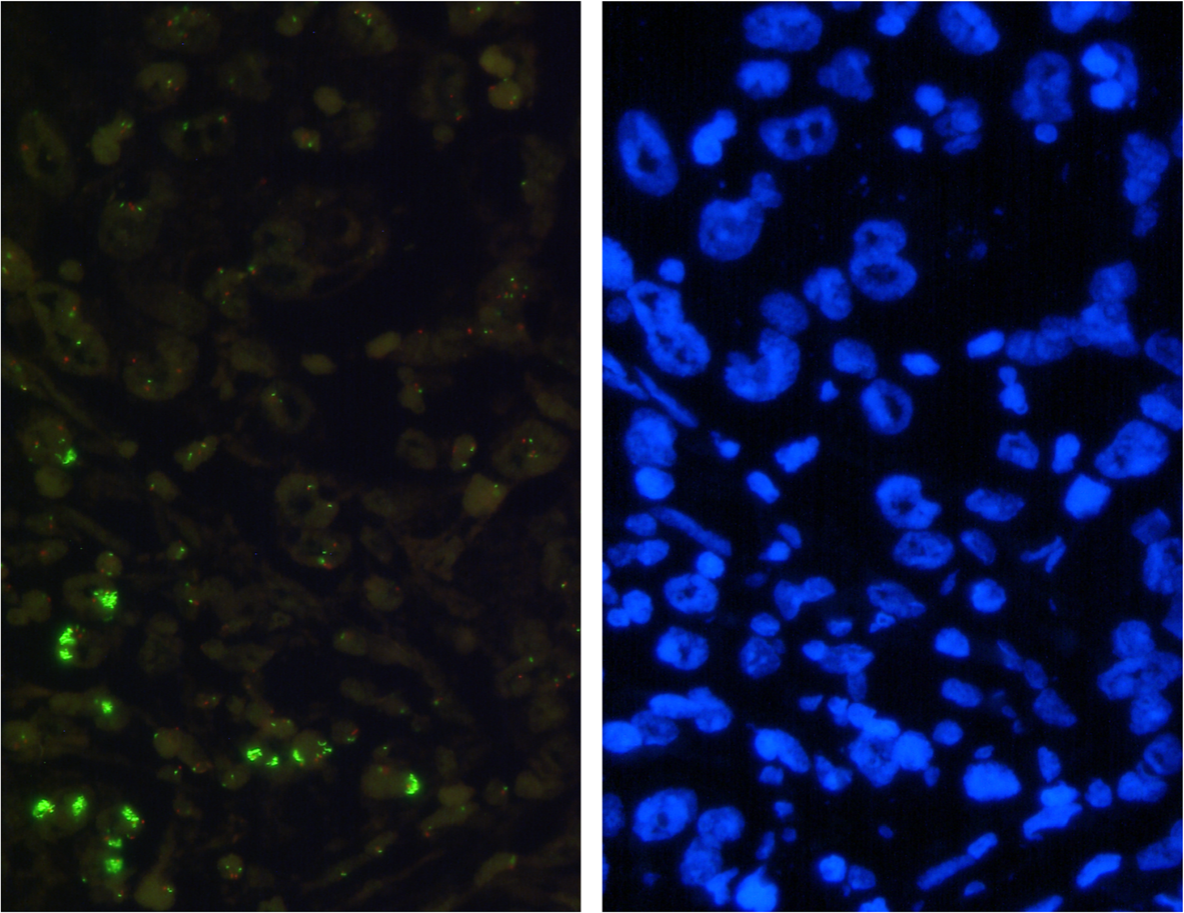
Intratumoral heterogeneity of *KRAS* amplification (*KRAS* FISH, 63x). Left: Green cluster signals: *KRAS* locus (=amplification). Red signals: Centromer region of chromosome 12. Right: nuclear counterstaining with DAPI

Gastric adenocarcinomas showed a varying homogeneity of *KRAS* amplified tumor clones within the tumor. *KRAS* amplified clones were found next to *KRAS* non-amplified tumor cell populations (see Fig. 3). All tumors amplified on TMA slides either representing primary tumors or metastasis were also analyzed on large scale slides. Heterogeneity was observed in 14 of 27 (51.9%) amplified tumors taking together heterogeneity within the primary tumor and those 3 cases with only *KRAS* amplified metastasis. In six cases (out of 14 *KRAS* amplified tumors with lymph node metastases) we could detect *KRAS* amplification in regional lymph node metastases but not in the primary available parts of the gastric tumor on TMA. We then analyzed further tumor parts of the stomach on large scale slides and were finally able to secure *KRAS* amplified clones in the primary tumor in three of the six cases (see discussion). Conversely, a primary tumor with *KRAS* amplification was found which was not found in regional lymph node metastases. The analyzed large scale of the primary tumor revealed a heterogenous distribution of *KRAS* amplified tumor cells in this case. The remaining 13 of 27 cases showed a homogeneous distribution of *KRAS* amplified carcinoma cells.

### *KRAS* amplification and KRAS protein expression

**Fig. 4:**
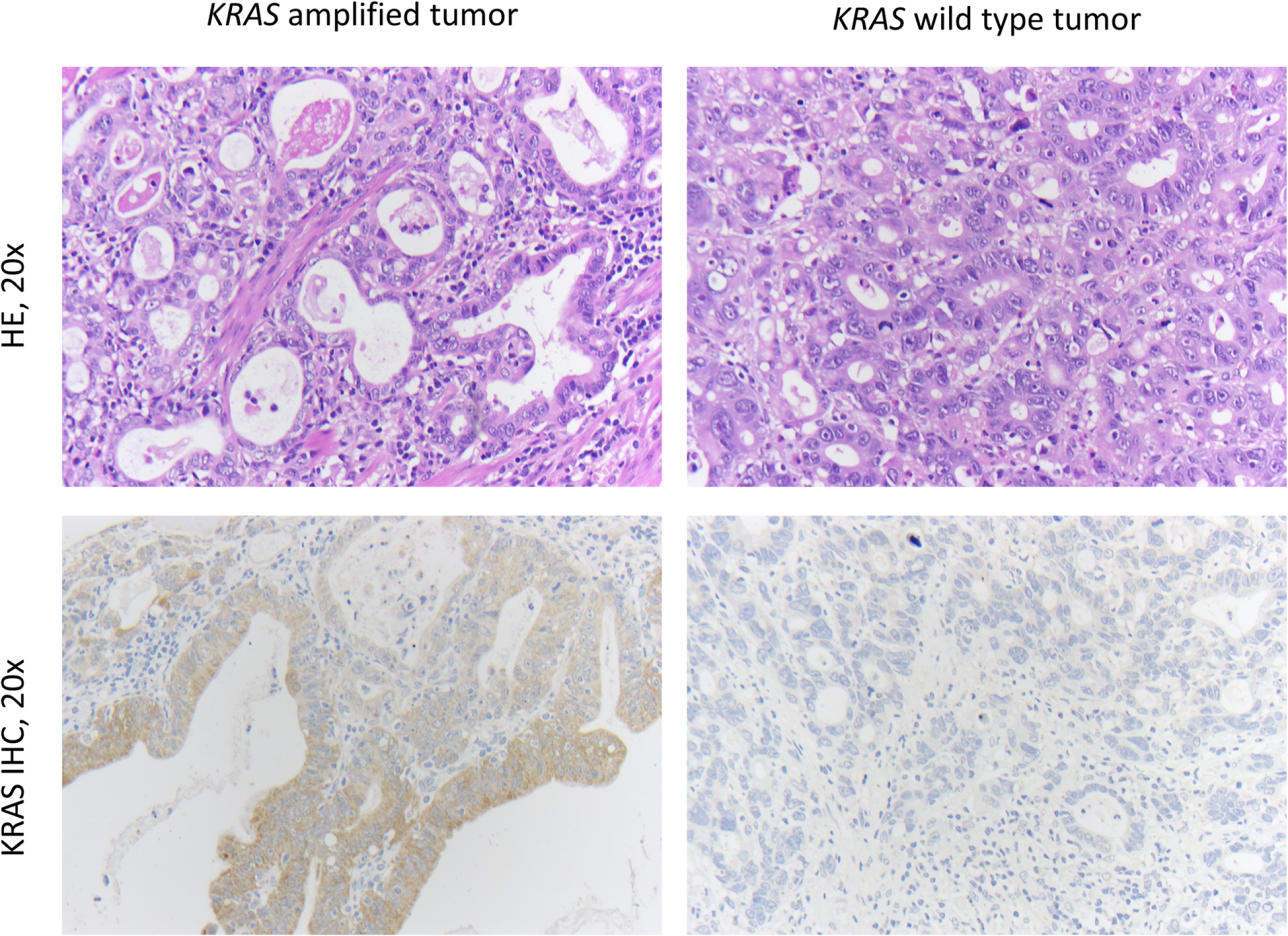
HE- and KRAS (IHC) staining in primary gastric adenocarcinomas. Left side: tumor sample with *KRAS* amplification. Right side: tumor sample without *KRAS* amplification

All tumors that harbored *KRAS* amplification detected by FISH analysis also expressed KRAS protein visualized by immunohistochemistry (Fig. 4). In 37 of 136 (27.2%) analyzed *KRAS* non-amplified tumor samples KRAS protein expression was observed.

## Discussion

Few studies exist that consider the importance of *KRAS* amplifications in gastric carcinoma [11, 16]. These studies, which analyzed Asian patient groups, showed, among other things, that *KRAS* amplification occurs largely mutation-exclusive and only in exceptional cases with simultaneous activating mutations of *KRAS*. These data are consistent with the results reported by the TCGA [8].

The *KRAS* amplification leads to an expression enhancement of the KRAS protein and to an activation of downstream partners (e.g. RAF, MEK). In addition, several studies reported for different cancer entities that *KRAS* amplified tumors are associated with poor prognosis [9, 10].

Nothing is known about the intratumoral heterogeneity of *KRAS* amplification in gastric adenocarcinomas and its importance and prognostic relevance in a large Caucasian patient population. Asian and Non-Asian patient populations differ in various aspects such as risk profiles of tumorigenesis, metabolic and genomic differences [17, 18].

In comparison to the TCGA data we found a slightly lower amplification frequency of *KRAS* in our collection of 582 patients (5.8% vs. 7.1%). We confirm the statistically significant unfavorable prognosis of *KRAS* amplified tumors in cases of primarily resected, non-neoadjuvantly treated tumors. We no longer see this prognostic effect in the group of neoadjuvantly treated and secondarily operated patients.

It is well documented that the group of so-called “minor responders” to a neoadjuvant therapy (= more than 10% vital tumor residual in the surgical specimens) has a significantly worse prognosis than the group of “complete responders” (= no vital tumor remnant) [19, 20]. Therefore, it is worth noting that in this subgroup only tumor tissue of “minor responders” was analyzable.

We explain our findings by the already poor overall prognosis of the patient cohort that cannot be further degraded measurably by the amplification of *KRAS*.

Nothing is known about the possible intratumoral heterogeneity of *KRAS* amplified tumor cell clones in gastric adenocarcinoma. This work demonstrates the relevance of the heterogeneity of *KRAS* amplified tumor clones in gastric carcinoma for the first time. This is particularly possible with the method used (FISH). In six cases we found *KRAS* amplification only in regional lymph node metastases, but not in the corresponding tissue of the primary tumor represented in the TMA. In three cases we were able to detect small *KRAS* amplified tumor cell populations in the primary tumor on additionally analyzed large scale slides of the primary tumor. This can be interpreted that *KRAS* amplified tumor clones show a special affinity for lymphogenic metastasis. This is underlined by the unfavourable prognostic relevance of *KRAS* amplification. In the three cases in which we were not able to detect a *KRAS* amplified tumor clone in the primary tumor (but with the detection of regional, *KRAS* amplified lymph node metastases), we assume that we could not find this clone because we did not have the entire primary tumor tissue but only parts for analysis. We consider the alternative that *KRAS* amplification only manifest itself in the course of lymph node metastasis to be unlikely.

Intratumoral heterogeneity of *KRAS* amplification should be taken into consideration for possible future therapeutic interventions on *KRAS*-amplified tumors and could be an argument for a re-determination of the amplification-status of the metastasis if the analyzable primary tumor fraction did not show any amplification (for example in a biopsy involving only small proportions of the tumor).

We also investigated the extent to which *KRAS* amplification correlates with the expression of the protein. All amplified tumors also show a corresponding protein expression. Therefore we confirm the results of other studies [11, 16]. KRAS protein expression was also observed in *KRAS* non-amplified tumors. Therefore KRAS immunohistochemistry is not suitable to screen for *KRAS* amplification.

According to the results of TCGA, gastric carcinomas can be divided into four major molecular subtypes, as outlined in the introduction. In our collection of tumors we find a divergent composition (see Table 2). However, in agreement with a previous publication [11], we found a marked accumulation of *KRAS* amplified tumors in the group of so-called chromosomal instable tumors (CIN) in almost 90%. No single microsatellite-unstable or EBV-associated carcinoma also shows *KRAS* amplification. The enrichment of *KRAS* amplified tumors in the CIN subgroup is consistent, as it also has increased gene amplifications such as *FGFR2* or *ERBB2*. We found a single tumor with a co-amplification of *ERBB2* and *KRAS*.

For esophageal and gastric adenocarcinomas it has been reported that high-level amplifications of *KRAS* resulted in a relevant copy number increase of the wild-type *KRAS* gene in the absence of coding mutations. This leads to an increased expression of KRAS protein with downstream activation, that can be influenced therapeutically [11, 21–23].

Our study has some limitations. This retrospective analysis only included tumor tissue from a single tumor center. We cannot make any reliable statements on the status of the primary tumor in the group of neoadjuvant treated patients. In this patient cohort, we only considered an accumulation of minor responders for neoadjuvant therapy, as in the group of complete responders no tumor material is available for analysis.

## Conclusion

In summary, we present the importance of *KRAS* amplification in gastric carcinoma in a large cohort of 582 Caucasian patients. While activating *KRAS* mutations are well characterized in various carcinomas, there is little reliable data on the relevance of *KRAS* amplification in gastric carcinoma. We confirm the unfavorable prognosis of *KRAS* amplified tumors reported by other studies for (Asian) patient groups. This study reveals intratumoral heterogeneity of *KRAS* amplification and demonstrates the correlation of gene amplification and protein expression. Despite the heterogeneous distribution of *KRAS* amplified tumor clones, *KRAS* amplified gastric adenocarcinomas represent a therapeutically highly interesting tumor subgroup.

Future prospective analyzes are necessary, which not only consider the usual *KRAS* mutations but also *KRAS* amplifications.

## Data Availability

The datasets used and analysed during the current study are available from the corresponding author on reasonable request.

## Abbreviations

CIN: Chromosomal instable tumors
DAPI: 4′,6-diamidino-2-phenylindole
EBV: Epstein-barr virus
FGFR2: Fibroblast growth factor receptor 2
FISH: Fluorescence-in-situ-hybridization
GCGC: Gastrointestinal cancer group cologne
GS: Genomically stable tumors
GTP: Guanosine triphosphate
Her2/neu and ERBB2: Human epidermal growth factor receptor 2; erythroblastic oncogene B
IHC: Immunohistochemistry
KRAS: Kirsten rat sarcoma viral oncogene
MAPK: Mitogen-activated protein kinase
MEK: MAPK/ERK kinase
MSI: Microsatellite unstable tumors
SHP2: Src homology 2 phosphotyrosyl phosphatase
SOS1/2: Son of sevenless homolog gene 1 / 2
TCGA: The Cancer Genome Atlas
TMA: Tissue microarray
ToGa trial: Trastuzumab for Gastric Cancer

## References

[1] Ferlay J, Soerjomataram I, Ervik M, Dikshit R, Eser S, Mathers C, Rebelo M, Parkin D, Forman D, Bray F: Cancer incidence and mortality worldwide: IARC cancerbase. Globocan 2012 v10 2012, 11. 10.1002/ijc.29210 25220842

[2] Bray F, Ferlay J, Soerjomataram I, Siegel RL, Torre LA, Jemal A (2018). Global cancer statistics 2018: GLOBOCAN estimates of incidence and mortality worldwide for 36 cancers in 185 countries. CA Cancer J Clin.

[3] Parsonnet J, Friedman GD, Vandersteen DP, Chang Y, Vogelman JH, Orentreich N, Sibley RK (1991). Helicobacter pylori infection and the risk of gastric carcinoma. N Engl J Med.

[4] Bornschein J, Selgrad M, Warnecke M, Kuester D, Wex T, Malfertheiner P (2010). H. Pylori infection is a key risk factor for proximal gastric cancer. Dig Dis Sci.

[5] Van Cutsem E, Sagaert X, Topal B, Haustermans K, Prenen H (2016). Gastric cancer. Lancet.

[6] Van Cutsem E, Bang Y-J, Feng-Yi F, Xu JM, Lee K-W, Jiao S-C, Chong JL, López-Sanchez RI, Price T, Gladkov O (2015). HER2 screening data from ToGA: targeting HER2 in gastric and gastroesophageal junction cancer. Gastric Cancer.

[7] Cho EY, Park K, Do I, Cho J, Kim J, Lee J, Kim S, Kim K-M, Sohn TS, Kang WK (2013). Heterogeneity of ERBB2 in gastric carcinomas: a study of tissue microarray and matched primary and metastatic carcinomas. Mod Pathol.

[8] Network CGAR (2014). Comprehensive molecular characterization of gastric adenocarcinoma. Nature.

[9] Birkeland E, Wik E, Mjøs S, Hoivik E, Trovik J, Werner HMJ, Kusonmano K, Petersen K, Ræder MB, Holst F (2012). KRAS gene amplification and overexpression but not mutation associates with aggressive and metastatic endometrial cancer. Br J Cancer.

[10] Wagner PL, Stiedl A-C, Wilbertz T, Petersen K, Scheble V, Menon R, Reischl M, Mikut R, Rubin MA, Fend F (2011). Frequency and clinicopathologic correlates of KRAS amplification in non-small cell lung carcinoma. Lung Cancer.

[11] Wong GS, Zhou J, Liu JB, Wu Z, Xu X, Li T, Xu D, Schumacher SE, Puschhof J, McFarland J (2018). Targeting wild-type KRAS-amplified gastroesophageal cancer through combined MEK and SHP2 inhibition. Nat Med.

[12] Simon R, Mirlacher M, Sauter G (2004). Tissue microarrays. Biotechniques.

[13] Helbig D, Ihle MA, Pütz K, Tantcheva-Poor I, Mauch C, Büttner R, Quaas A (2016). Oncogene and therapeutic target analyses in atypical fibroxanthomas and pleomorphic dermal sarcomas. Oncotarget.

[14] Loeser H, Waldschmidt D, Kuetting F, Heydt C, Zander T, Plum P, Alakus H, Buettner R, Quaas A (2017). Copy-number variation and protein expression of DOT1L in pancreatic adenocarcinoma as a potential drug target. Mole Clin Oncol.

[15] Valtorta E, Misale S, Sartore-Bianchi A, Nagtegaal ID, Paraf F, Lauricella C, Dimartino V, Hobor S, Jacobs B, Ercolani C (2013). KRAS gene amplification in colorectal cancer and impact on response to EGFR-targeted therapy. Int J Cancer.

[16] Deng N, Goh LK, Wang H, Das K, Tao J, Tan IB, Zhang S, Lee M, Wu J, Lim KH (2012). A comprehensive survey of genomic alterations in gastric cancer reveals systematic patterns of molecular exclusivity and co-occurrence among distinct therapeutic targets. Gut.

[17] Lin SJ, Gagnon-Bartsch JA, Tan IB, Earle S, Ruff L, Pettinger K, Ylstra B, Van Grieken N, Rha SY, Chung HC (2015). Signatures of tumour immunity distinguish Asian and non-Asian gastric adenocarcinomas. Gut.

[18] Chuah B, Goh BC, Lee SC, Soong R, Lau F, Mulay M, Dinolfo M, Lim SE, Soo R, Furuie T (2011). Comparison of the pharmacokinetics and pharmacodynamics of S-1 between Caucasian and east Asian patients. Cancer Sci.

[19] Cunningham D, Allum WH, Stenning SP, Thompson JN, Van de Velde CJ, Nicolson M, Scarffe JH, Lofts FJ, Falk SJ, Iveson TJ (2006). Perioperative chemotherapy versus surgery alone for resectable gastroesophageal cancer. N Engl J Med.

[20] Hölscher AH, Drebber U, Schmidt H, Bollschweiler E (2014). Prognostic classification of histopathologic response to neoadjuvant therapy in esophageal adenocarcinoma. Ann Surg.

[21] Dulak AM, Schumacher SE, van Lieshout J, Imamura Y, Fox C, Shim B, Ramos AH, Saksena G, Baca SC, Baselga J (2012). Gastrointestinal adenocarcinomas of the esophagus, stomach, and colon exhibit distinct patterns of genome instability and oncogenesis. Cancer Res.

[22] Das K, Gunasegaran B, Tan IB, Deng N, Lim KH, Tan P (2014). Mutually exclusive FGFR2, HER2, and KRAS gene amplifications in gastric cancer revealed by multicolour FISH. Cancer Lett.

[23] Chen Y, McGee J, Chen X, Doman TN, Gong X, Zhang Y, Hamm N, Ma X, Higgs RE, Bhagwat SV (2014). Identification of druggable cancer driver genes amplified across TCGA datasets. PLoS One.

